# Supplementary material for: Preparation of High Refractive Index Composite Films Based on Titanium Oxide Nanoparticles Hybridized Hydrophilic Polymers
Source: Nanomaterials (Basel). 2019 Apr 2;9(4):514. doi: 10.3390/nano9040514 (PMC6523180; doi:10.3390/nano9040514)
Supplement: Supplementary file 1 [file nanomaterials-09-00514-s001.pdf]

Supplementary Materials

Synthesis of Poly(*N*-hydroxyethylacrylamide), *p*HEAAm

Synthetic procedure

Poly[*N*-hydroxyethylacrylamide] (*p*HEAAm) was synthesized by free radical polymerization of *N*-hydroxyethylacrylamide (HEAAm, KJ Chemicals Corporation, Tokyo, Japan) in methanol. Briefly, 50 g of HEAAm was dissolved in methanol in a three-necked round-bottom flask. Oxygen was eliminated by bubbling of nitrogen gas for 30 min under stirring. 50.0 mg of 2,2'-Azobis(isobutyronitrile) (FUJIFILM Wako Pure Chemical Corporation (Osaka, Japan)) was added to the solution as an thermal initiator. Then the flask was then placed in an oil bath thermo-stated at 60 °C. After 1h, the solution was dropped into amount of acetone. The precipitate was dried in vacuum and again dissolved in methanol for washing and then precipitated again in excess acetone.

Yeild = 83%.

Characterization

Fourier transform infrared spectroscopy (FT-IR)
Powder samples were prepared by dispersing the polymers in KBr, and compressing the mixture to form disks. FTIR spectrum of *p*HEAAm was obtained by a FT-IR 4100 spectrometer (JASCO, Japan).

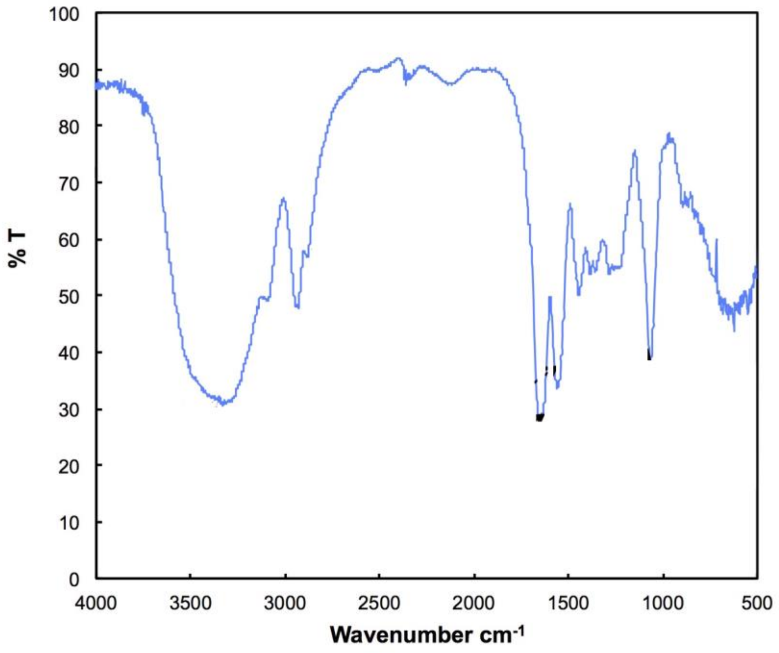

FT-IR spectrum of *p*HEAAm.

Around 3000 cm^{-1}: O–H (stretching) and N–H (stretching)
2931 cm^{-1}, 2884 cm^{-1}: C–H (stretching)
1650 cm^{-1}: C=O (stretching)
1558 cm^{-1}: N–H (bending)
1067 cm^{-1}: alcoholic C–O (stretching)

Size exclusion chromatography (SEC)

The weight average (M_w) molecular weight, and polydispersity indexes (PDI) of the prepared polymers were determined by size exclusion chromatography (SEC) with Shodex Asahipak GF- 7M HQ column (Showa Denko K. K., Japan) using dimethylformamide containing 0.01 M lithium chloride as eluent. Polystyrene standards were used as calibration standards.

*p*HEAAm

$M_w = 5.8 \times 10^5$
PDI = 2.87
